# CADASIL presenting as late‐onset mania with anosognosia

**DOI:** 10.1002/ccr3.2594

**Published:** 2019-12-08

**Authors:** Manik Uppal, Dora Kanellopoulos, Nabil Kotbi

**Affiliations:** ^1^ Weill Cornell Medical College New York Presbyterian Hospital New York NY USA

**Keywords:** anosognosia, bipolar disorder, CADASIL, personality change

## Abstract

The diagnosis of cerebral autosomal dominant arteriopathy with subcortical infarcts and leukoencephalopathy (CADASIL) should be considered in patients with late‐onset personality change and mania. However, neuropsychological deficits precipitated by the disorder pose significant challenges to recognition and appropriate management of CADASIL in susceptible patients.

## Graphical abstract

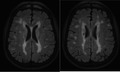

## 1 INTRODUCTION

Cerebral autosomal dominant arteriopathy with subcortical infarcts and leukoencephalopathy (CADASIL) manifests with progressive neurological disturbance and can present with neuropsychological deficits that pose significant challenges to clinical management. Here, we report a case of a female with CADASIL presenting with atypical initial manifestations of personality change, depression, and mania.

Cerebral autosomal dominant arteriopathy with subcortical infarcts and leukoencephalopathy (CADASIL) is an inherited cerebrovascular disease caused by a mutation of the Notch3 gene localized on chromosome 19.^1^ The disease is clinically characterized by a variable combination of migraine, recurrent transient ischemic attack or lacunar stroke, cognitive decline, psychiatric disturbances, and epilepsy.^2^, ^3^ Nearly, 34% of CADASIL patients have been found to have history of psychiatric disturbance attributed to the disease, and over 23% of this subset of patients feature mood disturbances like depression and bipolar disorder.^2^, ^4^ However, psychiatric disturbances most frequently occur later in disease progression and seldom represent the symptom at onset, accounting for only 6% of presenting pathology.

In order to highlight the diagnostic complexities inherent in the diagnosis of psychiatric manifestations of a primary neurological disorder, we present a case of atypical mania with psychotic features, secondary to progressive subacute cortical and subcortical infarcts due to CADASIL in a 64‐year‐old woman.

## 2 CASE

Ms A is a 64‐year‐old, married, domiciled, formerly employed woman who experienced insidious onset of worsening thought disorder, tangential thinking, paranoia, and verbal aggression toward her family starting 12 years prior to the current admission. On admission, thought content was consistent with persecutory paranoid delusions, namely that her family intended to harm her.

Psychiatric history included new‐onset depressive symptoms 17 years prior to the current admission and, initially, was treated on an outpatient basis with bupropion. Due to prominent neurovegetative symptoms (eg, sadness, anhedonia, lethargy), she was subsequently treated off label with amphetamine/dextroamphetamine. Around approximately the same time period, when the patient was in her mid‐ to late 40s, she also developed new‐onset alcoholism.

Medical history was significant for traumatic brain injury six years prior to the current admission, as she sustained two subdural hematomas secondary to a major fall; however, there was no evidence of lasting neurologic injury per chart review. Five years prior to the current admission, the patient was evaluated for CADASIL following discovery of white matter changes on brain MRI consistent with the pattern commonly observed in CADASIL. Genetic analysis was subsequently performed on the Notch3 gene. Direct DNA sequencing revealed a nucleotide substitution of A (TAT) to G (TGT) resulting in a change from tyrosine to cysteine at codon 1021 in exon 19 and thus confirmed CADASIL diagnosis. Chart review of the diagnostic neurological workup indicated that the patient was guarded regarding her medical diagnosis and did not want her family members to accompany her to her appointments. When asked during the current admission, the patient exhibited limited insight into her medical illness, reporting that she was not definitively diagnosed with CADASIL.

**Figure 1:**
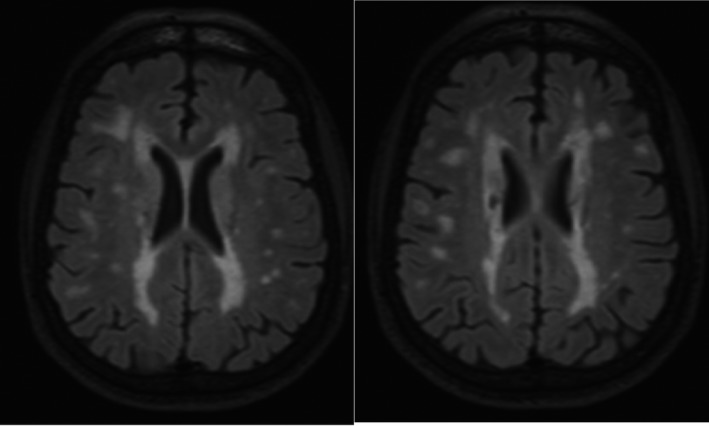
Axial planes of T2 FLAIR (fluid‐attenuated inversion recovery)‐weighted magnetic resonance imaging demonstrating multifocal subcortical and deep white matter hyperintensities involving both cerebral hemispheres, especially in the right frontal lobe, inferior parietal lobes, and bilateral external capsules

Brain MRI with and without contrast 10 months prior to admission revealed diffuse areas of patchy and confluent T2 hyperintense signal in the supratentorial and infratentorial white matter, including the bilateral anterior temporal lobes and external capsules. Chronic hemorrhagic infarctions were noted within the posterior right frontal lobe and inferior left parietal lobe (Figure 1). Multifocal areas of chronic microhemorrhage were observed within the brainstem and bilateral thalami. Subsequent brain MRI without contrast 5 months later further revealed new foci of susceptibility hypointensity indicative of chronic microhemorrhages in the lateral right cerebellar hemisphere and cerebellar vermis.

Clinical correlates around the same time of the brain MRI included worsening thought disorganization with paranoid ideation; the patient's primary care physician (PCP) also noted the onset of perseverative and persecutory thoughts regarding her family. The patient's PCP suggested the possibility of mania with psychosis possibly related to CADASIL. Until her current hospitalization, the patient herself maintained that she was unaffected by the CADASIL.

One month prior to the current admission, the patient experienced an acute exacerbation of paranoid ideation involving members of her extended family plotting to do her harm. She became verbally aggressive, exhibiting pressured speech and thought disorganization. She was initially admitted to a local hospital and treated with bupropion. Adderall was discontinued, and the patient was released to the care of her family; she discontinued bupropion when discharged. Four days later, she experienced a severe manic episode with mood‐congruent psychotic symptoms, including prominent agitation, flight of ideas, and emotional lability.

During the current inpatient psychiatric hospitalization, physical examination, EKG, laboratory examination results (comprehensive metabolic panel, urinalysis, and lipid profile) were normal. Her CBC was remarkable for mild anemia, and follow‐up testing was remarkable for anisocytosis, consistent with a prior diagnosis of autoimmune hemolytic anemia.

Ms A also underwent extensive neuropsychological testing. Results were not consistent with dementia, but rather were notable for deficits mainly in executive functions (eg, cognitive inhibition, cognitive flexibility, perseverative tendencies), as well as working memory, processing speed, and word retrieval. Learning and memory functions were well within normative limits. There was no significant decline in cognitive functions when compared to a prior evaluation 10 months earlier.

Symptoms of mania and psychosis responded to a treatment regimen of quetiapine (titrated up to 125 mg per day) and lithium carbonate 900 mg a day (level 0.84 mEq/L). Paranoid ideation decreased, and thought processes became increasingly linear and goal‐directed, though she remained perseverative. Furthermore, insight improved and the patient began to understand her psychiatric illness as a manifestation of CADASIL. Psychoeducation with the patient and her family helped to improve their communication. The patient was ultimately discharged to her family and agreed to work with an outpatient psychiatrist for follow‐up care.

## 3 DISCUSSION

This case report demonstrates the challenges of appreciating the neuropsychiatric manifestations of CADASIL, as well as the barriers to timely and appropriate psychiatric care. Although the prevalence of mood disorders and mania as presenting symptoms of CADASIL has been characterized,^4^ the diagnosis and treatment of the disease is often delayed when it first presents with psychiatric symptoms. This delay has profound consequences, as it frequently leads to poor psychosocial outcomes for CADASIL patients.

CADASIL varies widely in its clinical presentation, with migraines and transient ischemic attacks being the most commonly reported initial symptoms in patients aged 50.^5^, ^6^ Nearly, 24% of CADASIL patients have been found to have history of mood disturbance attributed to the disease, with around 88% presenting with major depression and between 5% and 8% with bipolar disorders or mania.^2^, ^4^

Several case studies describe mood disorders and personality change as initial manifestations of CADASIL. Gamakaranage et al described a case of an individual with CADASIL who presented with personality changes involving mood swings, aggressive behavior, and cognitive deficits, though the patient was deemed not to be manic.^7^ Leyhe et al reported the case of a patient who had experienced a hypomanic episode and became impulsive, verbally aggressive, irritable, and sexually preoccupied.^8^ There are also several previous reports describing patients with CADASIL presenting with mania.^9^, ^10^, ^11^ However, only the two most recent cases included descriptions of neuropsychiatric testing. Park et al described the case of a 53‐year‐old woman who experienced 5 years of progressive personality change until presenting with manic symptoms and pronounced deficit in attention and executive function, but intact memory.^10^ In a recent case report, a 62‐year‐old woman presented with mania, and deficits in memory and visuospatial reasoning, but intact attention and orientation.^11^ Notably, in each of these cases, diagnoses of CADASIL were made following the presentation of psychiatric symptoms.

Our case study extends on previous case literature in several aspects. First, while the patient's germline Notch3 mutation has previously been identified as a variant responsible for causing CADASIL, it has not been associated with psychiatric symptoms. Previous reports have instead linked the mutation to migraine and visual disturbance.^12^, ^13^ Second, our report illustrates the barrier posed by the patient's anosognosia regarding CADASIL in pursuing effective therapy for her psychiatric disturbance. Furthermore, unlike prior case reports, our patient was under active neurologic follow‐up for CADASIL at the time of presentation. However, her limited insight and paranoia prevented her from receiving adequate care from both her neurologist and psychiatrist.

Further, while previous cases have advocated for the exploration of organic causes like CADASIL in patients that exhibit late‐onset personality change,^10^ our case highlights the difficulty of appreciating insidious personality change in such patients. Our patient's progressive thought disorganization and paranoia toward her family undermined her established psychosocial support. Her unwillingness to share her medical history with her family, coupled with her limited insight regarding her diagnosis, prevented her from receiving adequate care for her neuropsychiatric decline. The cost of delayed or inappropriate care can be substantial, as our patient suffered marked occupational and familial dysfunction as a result of her illness, and coped with worsening alcoholism. As CADASIL is hypothesized to be an underdiagnosed disease, its manifestations and externalities even in diagnosed patients are likely to be underappreciated by the vast majority of clinicians.^5^, ^8^

## 4 CONCLUSION

The presentation of acute mania in the setting of CADASIL is an infrequent psychiatric manifestation of the disease. While prior studies have recommended the possibility of CADASIL diagnosis in patients with late‐onset personality change and mania, our case highlights the difficulties of recognition and appropriate management of CADASIL in susceptible patients.

## CONFLICT OF INTEREST

None Declared.

## AUTHOR CONTRIBUTIONS

MU: contributed to the conception and design of this study, and was involved in the patient treatment and interpretation of the treatment data. MU drafted the manuscript, provided critical revisions, and approved the final version of the manuscript for submission. DK: contributed to the conception and design of this study, and was involved in the patient treatment and interpretation of the treatment data. DK assisted in drafting the manuscript, provided critical revisions, and approved the final version of the manuscript for submission. NK: contributed to the conception and design of this study, was involved in the patient treatment and interpretation of the treatment data, and approved the final version of the manuscript for submission.
